# Early Life Exposure to Deltamethrin Impairs Synaptic Function by Altering the Brain-Derived Extracellular Vesicle Proteome

**DOI:** 10.1016/j.mcpro.2024.100902

**Published:** 2024-12-31

**Authors:** Leandra Koff, Jessica Di Re, Subhash Chand, Yosef Avchalumov, Nghi M. Nguyen, Timothy J. Baumgartner, Aditya K. Singh, Nana A. Goode, Mate Marosi, Lance M. Hallberg, Bill T. Ameredes, Thomas A. Green, Sowmya V. Yelamanchili, Gurudutt Pendyala, Fernanda Laezza

**Affiliations:** 1 Department of Pharmacology and Toxicology, University of Texas Medical Branch, Galveston, Texas, USA; 2 NIEHS Environmental Toxicology Training Program, University of Texas Medical Branch, Galveston, Texas, USA; 3 Department of Anesthesiology, University of Nebraska Medical Center, Omaha, Nebraska, USA; 4 Inhalation Toxicology Core, University of Texas Medical Branch, Omaha, Nebraska, USA

**Keywords:** exosome, Hebbian plasticity, hippocampus, neurodevelopment, pyrethroid

## Abstract

Pyrethroid pesticides have been associated with neurodevelopmental disorders including attention-deficit hyperactivity disorder (ADHD) and autism spectrum disorder (ASD). While behavioral effects of pyrethroid exposure have been previously reported, the underlying mechanisms remain unclear. Here, we hypothesized that exposure to deltamethrin (DM), a widely used pyrethroid pesticide known for its neurotoxicity during early developmental stages, induces brain dysfunction through alterations in brain-derived extracellular vesicle (BDEV) signaling. Using a well-established rodent model of early life DM exposure within the recommended no observable effect level, we isolated BDEVs from postnatal 30-day-old vehicle-exposed (control) and DM-exposed mice using a differential sucrose density gradient. Following ZetaView nanoparticle tracking and electron microscopy characterization, quantitative mass spectrometry-based proteomics revealed 89 differentially expressed proteins (DEPs) in BDEVs from DM exposed animals compared to control BDEVs. Bioinformatic analysis identified convergence of DEPs on pathways associated with mitochondrial function and synaptic plasticity. PKH67-green conjugated BDEVs derived from either control or DM-exposed mice were bilaterally injected intracerebroventricularly into naive adult mice, and the brain distribution of labeled BDEVs was verified prior to extracellular field recording experiments. Strikingly, long-term potentiation (LTP) at CA3-CA1 hippocampal synapses, a functional correlate of learning and memory, was intact in control BDEVs but absent in naive mice receiving BDEVs from DM exposed mice. Notably, exogenously delivering LRRTM1, one of the DEPs found in DM BDEVs, disrupts synaptic transmission in CA1 neurons consistent with impaired LTP. Thus, differentially regulated signaling in BDEVs represents a novel mechanism of DM neurotoxicity.

## Highlights

• Early life DM exposure alters brain derived extracellular vesicle (BDEV) proteome.
• Key altered proteins are within the pathways associated with synaptic function.
• DM-derived BDEVs severely disrupt hippocampal long-term potentiation (LTP) in males.
• Proteome dysregulation within the BDEVs is one way DM neurotoxicity is presenting.

## Graphical Abstract

**Figure:**
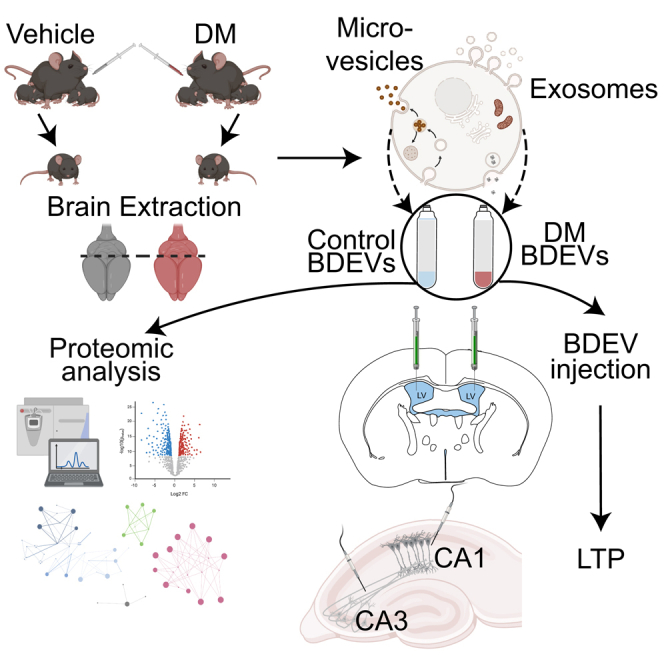

## In Brief

Early life exposure to deltamethrin (DM) changes the protein composition of brain-derived extracellular vesicles (BDEVs), impacting synaptic function pathways. Intracerebroventricular injection of DM-altered BDEVs into naive adult mice significantly disrupts hippocampal long-term potentiation (LTP) in response to high-frequency stimulation. This proteome dysregulation in BDEVs highlights a potential mechanism for DM toxicity in the brain.

Deltamethrin (DM) is a pyrethroid pesticide with growing use in agricultural and household settings, replacing the highly toxic organophosphates (1, 2, 3). Despite its perceived safety, epidemiological studies and rodent exposure models have indicated that DM and other pyrethroid pesticides can lead to severe neurotoxic effects throughout development and adulthood (4, 5, 6, 7). Factors contributing to the higher risk of DM toxicity in early life exposure compared to adults include the increased permeability of the blood-brain barrier to toxins during development and the ability of DM and other pyrethroids to permeate the placenta and reach the embryos and fetal brain during pregnancy (8, 9). Interestingly, it has been shown that males are more vulnerable to the toxic effects of DM than females in the current understanding of how certain neurodevelopmental disorders manifest (10, 11, 12). Concerns have specifically arisen regarding the association between early life pyrethroid exposure and neurodevelopmental disorders such as attention-deficit hyperactivity disorder (ADHD) and autism spectrum disorder (ASD), and learning disabilities, and increased suicide attempts (6, 9, 13, 14).

Neuronal deficits consistent with the neuronal alterations in the striatum and in the hippocampus have been reported in rodents exposed to DM during development, resulting in cognitive and behavioral deficits (12, 14, 15). Yet, the mechanisms underlying neuronal dysfunction associated with these deficits remain unclear. Of particular interest is the hippocampus, which plays a pivotal role in regulating cognitive function and social behavior, which are both impaired in ADHD and ASD (16, 17, 18).

Brain-derived extracellular vesicles (BDEVs) encompass various membrane-bound nanovesicles, including exosomes, microvesicles, and apoptotic bodies (19) with sizes ranging from 50 to 1000 nm. They transport proteins, lipids, nucleic acids, and metabolites (20), acting as indicators of cellular conditions and facilitating intercellular signaling throughout the brain (21, 22). Several studies have reported changes in BDEVs protein content associated with brain diseases and demonstrated that these extracellular vesicles can convey transferable molecular signals that communicate distally and exogenously (23, 24, 25, 26). Here, we hypothesized that BDEVs derived from mice exposed to DM during early life could carry signals capable of altering synaptic function. Using high-throughput mass spectrometry-based proteomics, we identified 89 differentially expressed proteins (DEPs) in DM-derived BDEVs associated with pathways including mitochondrial and synaptic function. Notably, intracerebroventricular (ICV) injection of fluorescently labeled BDEVs into adult naive mice induced a loss of long-term potentiation (LTP) in the hippocampus of animals receiving BDEVs from DM exposed mice. Thus, alterations in the BDEVs proteome signaling suggest a potential mechanism of DM synaptic toxicity, which could contribute to previously reported cognitive and behavioral deficits associated with neurodevelopmental disorders. Additionally, these findings further corroborate the pivotal role of BDEVs in regulating synaptic function and serving as vehicles for carrying signals that contribute to endophenotypes in brain disorders.

## Experimental Procedures

### Experimental Design and Statistical Rationale

All analyses were performed in GraphPad Prism 9.0.0 (GraphPad Software). Normal distribution of data was examined before analysis and appropriate statistical tests were performed. A Mann–Whitney *U* test was used to analyze the effect of DM on average BDEV size and paired-pulse ratio. Due to differences in variance in samples, an unpaired *t* test with Welch’s corrections was used to analyze the effect of DM on BDEV concentration and on the slope change of field excitatory postsynaptic potentials (fEPSPs). A two-way ANOVA of the log-normalized data was used to determine the effect of DM on a range of particle sizes. In all cases, *p* < 0.05 was considered statistically significant.

A cutoff of *p* < 0.05 by *t* test and a fold-change of ≥1.5 was used to determine DEPs. The Cytoscape plug-in ClueGO was used for gene ontology (GO) analysis (27). GO enrichment analysis included biological processes and molecular functions. Canonical pathway analysis was performed using Ingenuity Pathway Analysis (IPA) software (Ingenuity Systems, Redwood City, www.ingenuity.com, accessed on 5 October 2023) by comparing the DEPs against known canonical pathways (signaling and metabolic) within the IPA database (28). The z-score in IPA was calculated as described in (28). A positive z-score extrapolated the activation state, while a negative z-score predicted the inhibition state. A −log (*p*-value) cutoff of 1.3 (*p*-value that was less than or equal to 0.05) was applied, by default, to show only significant canonical pathways.

### Animals

Mice were maintained on an inbred C57/BL6J background (greater than 10 generations of backcrossing to C57/BL6J). Wild-type C57/BL6J mice were purchased from Jackson Laboratory. All surgical and experimental procedures employed were approved by the University of Texas Medical Branch Institutional Animal Care and Use Committee under protocol 0903016D.

### Early Life Exposure Model

Early-life exposure to DM was performed as previously described with slight modifications (1, 11). 8-week-old wild-type C57/Bl6 (Jackson Laboratory) female mice (2 females per male) were introduced to males, at which point dams were orally exposed to 3 mg/kg/72 h DM (Sigma-Aldrich) or vehicle (Corn oil and peanut butter in a 2:1 ratio). This dose was chosen as it corresponds with the no observed adverse effects level (NOAEL) of 1 mg/kg/24 h set by the US Environmental Protection Agency (EPA). There were a total of 9 control dams and 10 DM dams. Dosing started the same day as pairing and continued throughout pregnancy and lactation until PND21 (weaning). Females were separated 14 days after pairing. If the dam did not give birth within 21 days of separation, it was excluded from the experiment.

### BDEV Isolation

At postnatal day 30 (PND30), approximately 10 days after exposure cessation, pups exposed to DM and vehicle were sacrificed by deep anesthesia using isoflurane and decapitated. Brains were extracted and dissected into cortex and striatum and then immediately frozen in liquid nitrogen vapor. Tissue from multiple animals (n = 2–4 animals) from a single litter were pooled together for a starting tissue weight of 100 to 200 mg before BDEV isolation. Biological sex was not considered as a variable. In total, tissue from 19 litters (9 control, 10 DM-exposed) were used. Three control and 4 DM litters were used for proteomics and TEM analysis; 3 control and 3 DM litters were used Zeta-view analysis (2 samples per litter); 3 control and 3 DM litters were used for follow-up Western Blot studies; and 3 control and 3 DM litters were used for BDEV labelling and subsequent intracerebroventricular (ICV) injection. Except for the fluorescently labelled BDEVs used for injections, isolated BDEV samples were used for multiple analyses.

BDEV isolations from mouse brains were carried out following our previously established protocols (16, 19, 20, 21). Briefly, frozen cortical brain tissue weighing around 100 to 200 mg was minced into smaller pieces and treated with 10 units/ml papain (P4762, Sigma-Aldrich) in 3.5 ml Hibernate A (NC0176976, Fisher Scientific) solution and rocked for 15 min at 37 °C. The reaction was quenched with a 6.5 ml cold Hibernate A solution containing antipain (A6191) and protease inhibitors (S8820). The homogenate was then sequentially centrifuged at 300*g* for 10 min, 2000*g* for 10 min, from which the supernatant was taken every time, and 10,000*g* for 30 min to remove cell debris, heavier membranous structures, and larger vesicles. The supernatant was filtered through a 0.20 μm syringe filter (09–754–13, Corning Fisher Scientific) and centrifuged at 100,000*g* for 60 min at 4 °C to pellet crude BDEV. The pellet was washed with 35 ml of cold particle-free PBS at 100,000*g* for 60 min at 4 °C. The crude BDEV pellet was then resuspended in 2 ml of 0.95 M sucrose solution and layered in between 0.60 M and 1.30 M sucrose layers. The sucrose gradient column comprising six gradients in 2 ml volume per step, starting at 2.0 M at the bottom to 0.25 M sucrose solution at the top (with a 0.35 M increment) was centrifuged at 200,000*g* for 16 h at 4 °C. From the top of the gradient, a 2 ml fraction was discarded. The next 6 ml was collected, diluted to a total volume of 35 ml with ice-cold particle-free PBS, and centrifuged at 100,000*g* at 4 °C for 60 min to collect purified BDEV pellets. BDEV pellets were finally suspended in 50 to 70 μl PBS particle-free with protease inhibitors and used for downstream analysis.

### Zeta View Analysis

Concentration and size distribution curves of the BDEV were carried out by Zeta View on n = 3 control and n = 3 DM litters, with 2 samples per litter. To measure size and concentration, 10 μl of BDEV were diluted to 1:100 to 1:5000 in particle-free PBS before measurements, according to the user manual. Data were analyzed using GraphPad.

### Transmission Electron Microscopy (TEM)

TEM was performed as described previously (19, 20) on n = 3 control and n = 4 DM litters. A 5 μl sample of BDEV was mixed with 45 μl of TEM fix buffer (2% paraformaldehyde, 2% glutaraldehyde, and 0.1 M phosphate buffer). A 10 μl drop of BDEV-buffer mix sample was spotted onto formvar/silicon monoxide coated 200 mesh copper grids (Ted Pella Inc.) and allowed to sit for 3 min. Before use, grids were glow discharged for 60 s at 20 μA with a GloQube glow discharge unit (Quorum Technologies). The excess solution was blotted off on Whatman filter paper, and the sample was allowed to dry for 1 min. Samples were negatively stained with NanoVan (Nanoprobes, New York, NY) and examined on a Tec-nai G2 Spirit TWIN (FEI, Hillsboro, OR) operating at an accelerating voltage of 80 kV. AMT digital imaging system was used to acquire images.

### Western Blotting

The BDEV protein preparation and western blotting have been done as described (20, 29, 30, 31). BDEVs from n = 3 control and n = 3 DM litters were used for western blotting. Briefly, the BDEV protein concentration was quantified using the Pierce BCA protein assay kit (Thermo Fisher Scientific). Proteins from respective samples (20 μg) were loaded in each NuPAGE 4 to 12% Bis-Tris gels (Invitrogen) lane. The tetraspanin (CD81) markers were run under non-reducing conditions. The Alix, Hsp-70, and beta-Actin BDEV-markers were run under reducing conditions. The proteins were transferred from the gel onto nitrocellulose membranes using the iBlot 2 Gel Transfer Device (Invitrogen). The nitrocellulose membranes were blocked in TBS SuperBlock buffer (Thermo Fisher Scientific) and were incubated overnight at 4 °C with the primary antibody (Supplemental Table S1) on a rocker. The HRP-conjugated secondary antibodies were incubated for 1.5 h at room temperature on a rocker (Supplemental Table S1). Blots were developed with 1:1 Chemiluminescent Substrate and Luminol/Enhancer (Azure Biosystems) and visualized using a c300 imaging system (Azure Biosystems). Images were quantified using ImageJ software.

### Proteomics Analysis

Protein quantification was performed using Pierce BCA protein assay (Thermo Scientific) and followed the procedure described in our earlier studies (20, 32). MS analysis was established and managed with a UNMC Mass Spectrometry Core. The analysis was based on the label-free quantitative mass spectrometry protocol. Specifically, 50 μg of protein per sample from control (n = 3) and DM (n = 4) litters was processed. Litters were used as biological replicates and included tissue from 2 to 4 animals per litter. Tissue was subjected to chloroform–methanol extraction to remove the detergent in each sample. Before mass spectrometric analysis, the protein pellet was resuspended in 100 mM ammonium bicarbonate and digested with MS-graded trypsin (ThermoFisher) overnight at 37 °C. A 1:100 ratio of trypsin was used for protein digestion. Samples were treated with 10 mM DTT at 56 °C for 30 min and alkylation was performed using 50 mM iodoacetamide at room temperature for 25 min.

The peptides were then cleaned using PepClean C18 spin columns (Thermo Scientific) and resuspended in 2% acetonitrile (ACN) and 0.1% formic acid (FA). Then, 500 ng of each sample was loaded onto trap column Acclaim PepMap 100 75 μm × 2 cm C18 LC Columns (Thermo Scientific), at a flow rate of 4 μl/min and then separated with a Thermo RSLC Ultimate 3000 (Thermo Scientific) on a Thermo Easy-Spray PepMap RSLC C18 75 μm × 50 cm C-18 2 μm column (Thermo Scientific) with a step gradient of 4 to 25% solvent B (0.1% FA in 80% ACN) from 10 to 130 min, and 25 to 45% solvent B for 130 to 145 min at 300 nl/min and 50 °C with a 180 min total run time.

The eluted peptides were then analyzed using a Thermo Orbitrap Fusion Lumos Tribrid (Thermo Scientific) mass spectrometer in a data-dependent acquisition mode to analyze eluted peptides. A complete survey scan MS (from m/z 350–1800) was acquired in the Orbitrap with a resolution of 120,000. The AGC target for MS1 was set as 4 × 10^5^, and the ion filling time was set as 100 ms. The most intense ions with charge states 2 to 6 were isolated in a 3 s cycle and fragmented using HCD fragmentation with 40% normalized collision energy and detected at a mass resolution of 30,000 at 200 *m/z*. The AGC target for MS/MS was set as 5 × 104 and the ion filling time was set at 60 ms; dynamic exclusion was set for 30 s with a 10 ppm mass window.

### Protein Identification

Protein identification was performed by searching MS/MS data against the SwissProt mouse protein database downloaded in October 2021, which included 17,748 reviewed entries, using the in-house PEAKS X + DB search engine (20, 29, 33). The search targeted total tryptic peptides and allowed for two missed cleavage sites. Carbamidomethylation of cysteine was set as a fixed modification. Protein N-terminus acetylation and methionine oxidation were included as variable modifications. The highest permitted fragment mass error was 0.02 Da, and the precursor mass tolerance threshold was set at 10 ppm. A false discovery rate (FDR) of ≤1% was used to calculate the significance threshold of the ion score. Progenesis QI proteomics 4.1 (Nonlinear Dynamics) was used for qualitative analysis as described in (34). Briefly, the program automatically selected one run to align the LC-MS runs with to account for retention time changes. Only peptide ions having at least three isotopic peaks and charge states +2, +3, and +4 were taken into consideration when creating a master list of characteristics that took retention time and *m/z* into account. The normalized ion intensities of each of a protein's nonconflicting peptides were considered when calculating the protein abundances.

### Bioinformatics Analysis

A cutoff of *p* < 0.05 by *t* test and a fold-change of ≥1.5 was used to determine DEPs. The Cytoscape plug-in ClueGO was used for gene GO analysis (27). GO enrichment analysis included biological processes and molecular functions. Canonical pathway analysis was performed using Ingenuity Pathway Analysis (IPA) software (Ingenuity Systems, www.ingenuity.com, accessed on 5 October 2023) by comparing the DEPs against known canonical pathways (signaling and metabolic) within the IPA database (28). The z-score in IPA was calculated as described in (28). An absolute z-score of ≥2 was considered significant. A positive z-score extrapolated the activation state, while a negative z-score predicted the inhibition state of the upstream. A −log (*p*-value) cutoff of 1.3 (*p*-value ≥0.05) was applied, by default, to show only significant canonical pathways.

### BDEV Labeling and Injection

BDEV were labeled with PKH67-green (PKH67Gl) fluorochromes with excitation (490 nm) and emission (504 nm) as per the manufacturer’s suggestions. Naïve 12-week-old WT C57/BL6 male mice were injected with BDEVs from either control or DM derived BDEVs at similar concentrations (Control 7 × 10ˆ11 particles per mL; DM: 4.5 × 10ˆ11 particles per mL). Injection coordinates used for ICV targeting were AP: −0.5, Lat: ± 1.0, DV: −2.2, according to the Paxinos atlas (35). AP coordinates were adjusted per individual mouse by measuring the distance between bregma and lambda and dividing this distance by the interaural distance published in Paxinos (3.8 mm). This value was multiplied by the AP coordinate (−0.5) to determine correct AP positioning. Bilateral injections were performed for a total of 4 μl (2 μl per side) for each animal at a rate of 0.2 μl per minute, which is the approximate flow rate of cerebral spinal fluid in the ventricles (36, 37, 38).

### Confocal Imaging

36 h after control BDEV injection, one animal was deeply anesthetized using isoflurane and euthanized to validate the BDEV injection and uptake. Following extraction, the brain was sectioned using a vibratome at 200 μm. Slices were rinsed once with cold PBS, followed by a 30-min fixation in 1% paraformaldehyde (diluted from 37% paraformaldehyde (Sigma-Aldrich), then rinsed 3 times with cold PBS. For colocalization experiments, slices were stained with a NeuN antibody (1:250, Synaptic System, Göttingen, Germany, catalog number 266 004) overnight at 4 °C, then with an isotype-specific antibody (1:200, Alexa 647, Thermo Fisher Scientific) at room temperature for 2 h. Slices were counterstained with DAPI for 5 min, then rinsed 3 times with cold PBS before mounting with ProLong Gold. Slices were imaged at the UTMB Optical Microscopy Core using a Zeiss LSM880 confocal microscope with a 63× oil objective (1.4 NA), and excitation lines at 405 nm (DAPI), 488 nm (PKH67Gl), and 633 nm (NeuN). Gain and offset were kept constant throughout imaging. To image the entire hippocampus, a series of tile scans (512 × 512) were taken and stitched using Zeiss ZEN microscopy software. CA1 zoomed images (1024 × 1024) were taken with the same laser, gain, and offset setting to visualize BDEV distribution. For colocalization experiments, images were taken in AiryScan mode and all acquisition parameters, including photomultiplier master gain (405 nm: 860, 488 nm: 880, 633 nm: 850), gain (for all channels: 1), and zoom (3.0) were kept constant throughout each batch of experiments. Z-stacks with 0.5 μm width were captured and analyzed using Fiji (NIH) by counting the number of exosomes within or near the NeuN-labeled neurons (Class I) and the exosomes at a distance from the neurons (Class II). The numbers were expressed as a percentage of the total exosomes per 3× zoom view.

### Extracellular Field Recordings

36 h after BDEV injection, field excitatory postsynaptic potentials (fEPSPs) from the stratum radiatum (CA1) of the hippocampus were recorded by stimulating Schaffer collaterals. Basal synaptic transmission was analyzed by generating stimulus/response curves for single as well as paired-pulse ratios of synaptic responses elicited with a 50 ms inter-stimulus interval. After obtaining a stable baseline, LTP was induced by tetanic stimulation (100 Hz, 1s). Following recordings, slices were fixed in 1% formaldehyde, and each was used to visually validate BDEV injection using an epifluorescence microscope.

### Whole-Cell Patch-Clamp Electrophysiology Recordings

For whole-cell patch-clamp electrophysiology recordings, acute hippocampal slice preparations were obtained as previously described (39, 40). During slice preparation, the tissue was kept in an ice cold and continuously oxygenated (95% O2/5% CO2) tris-based cutting solution comprised of the following salts: 125 mM NaCl; 2.5 mM KCl; 20 mM HEPES; 1.25 mM Na2HPO4; 30 mM NaHCO3; 20 mM HEPES; 25 mM glucose; 3 mM Na pyruvate; 5 mM Na ascorbate; 5 mM MgCl2 and 0.5 mM CaCl2 (pH = 7.4 and osmolarity = 300–310 mOsm; all salts purchased from Sigma-Aldrich). Following preparation of slices, they were immediately transferred into heated (37 °C) and continuously oxygenated tris-based cutting solution for 15 min. After 15 min, slices were transferred to heated (37 °C) and continuously oxygenated standard artificial cerebrospinal fluid (aCSF) comprised of the following salts: 125 mM NaCl; 2.5 mM KCl; 10 mM glucose; 1.2 mM MgCl2; 2.5 mM CaCl2; 25 mM NaHCO3; and 1.25 mM Na2HPO4 (pH = 7.4 and osmolarity = 300–310 mOsm; all salts were purchased from Sigma-Aldrich). Following an ample recovery period, slices were transferred to another bath containing heated (37 °C) and continuously oxygenated ACSF with the addition of vehicle (0.01% BSA, New England Biolabs) or 100 ng/ml LRRTM1(Biotechne Catalog # 4897-LR). Following a 1-h exposure period, slices were placed into the recording chamber, which was perfused with heated (37 °C) and continuously oxygenated aCSF at a flow rate of 150 ml/h. Spontaneous excitatory postsynaptic currents (sEPSCs) were subsequently recorded in visually identified CA1 pyramidal neurons. Recordings were performed using borosilicate glass pipettes containing the following internal solution: 145 mM K-gluconate; 2 mM MgCl2; 0.1 mM EGTA; 2.5 mM Na2ATP; 0.25 mM Na2GTP; 5 mM phosphocreatine; and 10 mM HEPES (pH = 7.2 and osmolarity = 290 mOsm; all salts were purchased from Sigma-Aldrich). After GΩ seal formation and entry into the whole-cell configuration, membrane capacitance and series resistance were compensated for as described above. After compensation, the amplifier was switched to I = 0 mode for 1 to 2 min to assess resting membrane potential of each cell. During this 1 to 2 min interval in I = 0 mode, a solution of 20 μM bicuculline (Tocris) was perfused to isolate sEPSCs. After assessment of resting membrane potential, cells were kept at a holding potential of −70 mV, and synaptic activity was recorded for 3 min sEPSC recordings were quantified and data was analyzed using GraphPad Prism 9.

## Results

### Early Life DM Exposure Does Not Affect Structural Characteristics of BDEVs

**Fig. 1:**
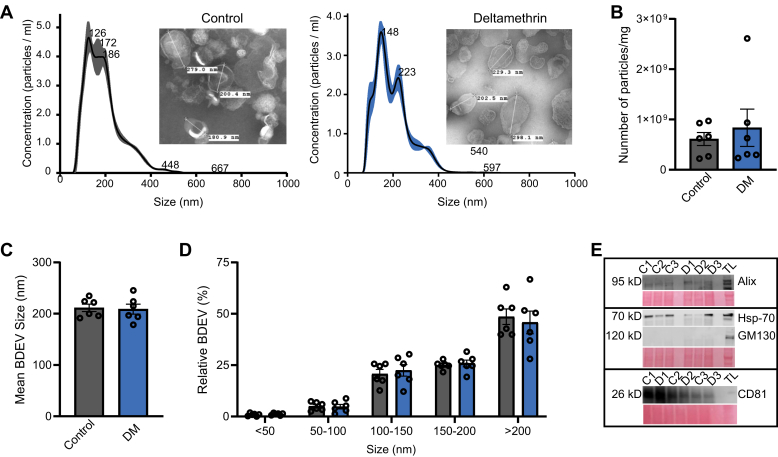
**Characterization of DM BDEVs.***A*, representative TEM and ZetaView analysis of Control and DM BDEV size and concentration. *B*, ZetaView nanoparticle tracking analysis shows no change in BDEV concentration between control or DM treated animals as determined by an unpaired *t* test following a Welch’s correction (n = 3 control and 3 DM litters, 2 samples per litter). *C*, The mean BDEV size is not altered between control and DM treated animals using an unpaired *t* test. *D*, relative size distribution is not altered by DM as determined by a two-way ANOVA, indicating that there is no change in different types of extracellular vesicles. *E*, Western blot analysis on BDEVs isolated from control and DM animals shows the expression of the positive markers Alix, Hsp-70, B-Actin, and CD81 both in control and DM BDEVs (n = 3 control and 3 DM litters).

To investigate the effect of early-life exposure to DM on BDEVs, we isolated BDEVs from offspring of dams that were exposed to either corn oil and peanut butter (vehicle, control) or 3 mg/kg DM in vehicle once every 3 days. This previously published dosing model was chosen to reduce maternal stress and mimic the NOAEL set by the EPA (41). BDEVs were isolated utilizing the ultracentrifugation method from the multiple sucrose gradients, as described in previous studies (29, 31, 42). BDEV concentration, mean size, and size distribution were characterized by TEM and analyzed by Zeta View (Fig. 1*A*). Vehicle BDEV and DM BDEV did not differ significantly in their concentrations (*p* = 0.58, n = 3 control and 3 DM litters, 2 samples per litter), sizes (*p* = 0.84), or relative percentages (*p* > 0.99; Fig. 1, *A*–*D*). To validate the purity of the isolated BDEVs, Western blot analysis was performed to confirm the presence of classical extracellular vesicle constituent proteins including the positive markers Hsp70 and CD81 and the absence of the marker GM130 (n = 3 control and 3 DM litters; Fig. 1*E*). Ponceau red staining was used for total protein normalization, as shown in both control and DM groups (Fig. 1*E*). Thus, developmental exposure to DM does not affect the overall characteristics of BDEVs.

### Early Life DM Exposure Alters the Proteomic Content of BDEVs

**Fig. 2:**
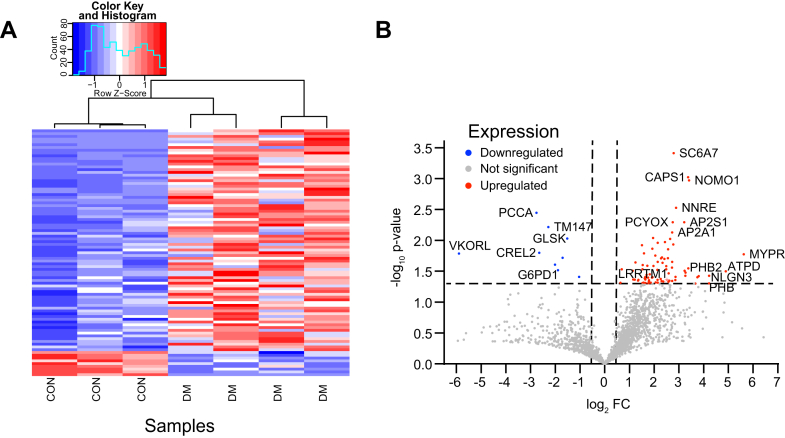
**DM exposure alters BDEV proteome.***A*, Heatmap showing the DEPs in the control (n = 3) *versus* DM treated (n = 4) litters. *B*, Volcano plot showing significantly (*p* < 0.05) changed proteins. Proteins of interest have been labelled.

We next sought to determine whether early-life exposure to DM induces alterations in the BDEV protein content. Therefore, BDEVs from both groups (n = 3 control and 4 DM litters) were subjected to high throughput quantitative mass spectrometry-based proteomics as described in our previous works (20, 29, 30, 31, 42, 43). A total of 2946 proteins were identified (Supplemental Table S2) from which a total of 2625 proteins were reproducibly quantified in all the replicates. From this pool, 89 were differentially expressed in the two groups based on a fold-change of greater than 1.5 with a *p* < 0.05 as depicted in the heatmap (Supplemental Table S3 and Fig. 2*A*). The volcano plot in Figure 2*B* depicts the proteins that were up (red) or down (blue). A total of 80 were upregulated, while nine were downregulated concerning the control group.

**Fig. 3:**
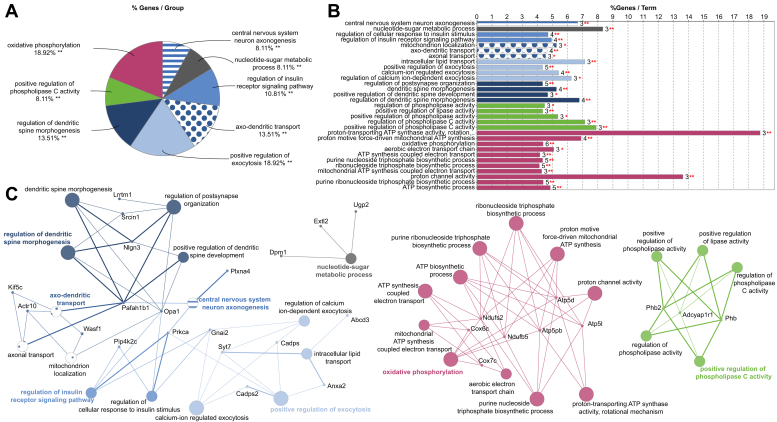
**Bioinformatic analysis of altered DM BDEV proteins**. *A*, percentage of genes per group mapped to biological processes. *B*, histogram of altered genes per term in ClueGo analysis. *C*, networks show the interaction of proteins and their biological process for the DEPs (Control n = 3 and DM treated n = 4) using ClueGo. The *asterisks* represent the group term *p*-value representing each category. ∗∗*p* < 0.01.

Further analysis of the DEPs was performed using GO analysis with the Cytoscape plug-in ClueGO (Fig. 3, *A*–*C*). The most enriched biological processes were oxidative phosphorylation and positive regulation of exocytosis, with 18.92% of gene GO terms associated with each of these processes (Fig. 3*A*). Some of the other biological processes enriched were mitochondrial functions, including ATP synthase activity, as well as regulation of dendritic spine morphogenesis, axodendritic transport, and phospholipase C (PLC) activity (Fig. 3, *A* and *B*). Figure 3*C* represents all the possible connections of the networks between the DEPs associated with GO terms and processes, which align with the Ingenuity Pathway Analysis (IPA; Supplemental Fig. S1). Of particular interest were terms associated with dendritic spine morphogenesis due to their critical role in synaptic plasticity, particularly in the form of long-term potentiation (LTP), which is believed to underlie learning and memory formation (44).

### Exogenously Delivered BDEVs From DM-Exposed Mice Carry Signals that Disrupt Synaptic Function

**Fig. 4:**
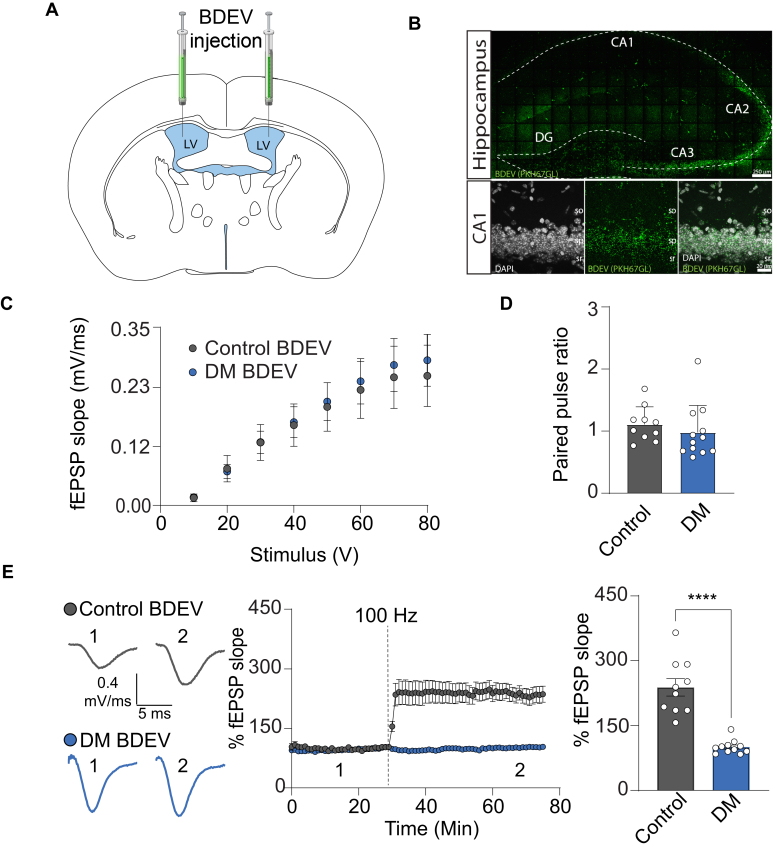
**DM BDEVs alter LTP but not PPF in naive mice.***A*, bilateral ICV injection of control or DM BDEVs into male naive mice. *B*, scan of hippocampus after ICV injection of BDEVs (*green*) and DAPI (*whit*e) with overlay of hippocampus anatomy (scale bar indicates 250 μm). Lower zoomed in images on the CA1 region show DAPI and BDEVs and then a merge of both channels (scale bar indicates 20 μm). *C*, fEPSP slope plotted as a function of stimulation intensity to assess basic synaptic responses in slices. *D*, PPF ratio comparison in CA1 of injected mice indicates that DM BDEVs do not alter presynaptic function as determined by an unpaired *t* test (control n = 10 and DM n = 12) *E*, representative traces from male control mice before (*black/left*) and after (*gray*) and male DM BDEV before (*black/right*) and after (*blue*) LTP induction by tetanic stimulation of Schaffer collaterals (100 Hz, 1s). Field excitatory postsynaptic potential (fEPSP) from the striatum radiatum (CA1) of the hippocampus were recorded for 30 min prior to tetanic stimulation from males injected with control BDEVs (*gray*) and DM BDEVs (*blue*). A total of 70 min was recorded and slope change was calculated for each slice. Then, analysis of fEPSP slope change indicates DM BDEV injection results in a loss of LTP in males as indicated by the lack of change in slope of fEPSPs following tetanic stimulation as determined by an unpaired *t* test with a Welch’s correction (control n = 10 and DM n = 12) ∗∗∗∗indicates *p* < 0.001 by unpaired *t* test.

The DEPs in BDEVs from DM exposed animals indicated alterations in proteins previously linked to synaptic function, including terms associated with synaptic function, such as dendritic spine morphology and axo-dendritic transport which are of particular interest given their role in LTP, a form of plasticity believed to underlie learning and memory formation (44). These perturbations are in line with previous findings that DM has a sex specific effect in males, in which exposure has been linked to long lasting effects on learning, memory, and numerous ADHD and ASD-related phenotypes (10, 11, 12). Hence, we hypothesize that content from BDEVs from DM exposed animals could contain signaling molecules that, if transferred exogenously to naïve animals, would be sufficient to alter synaptic function. To test this hypothesis, PKH67-green conjugated BDEVs derived from either control or DM exposed mice, were bilaterally intracerebroventricularly (ICV) injected via stereotaxic surgeries on naïve 12-week-old male mice (Fig. 4*A*). Similar to earlier reports, we found that 36 h after ICV injection, BDEVs achieved widespread dispersal throughout the brain, including the hippocampus and dentate gyrus, with concentrated uptake occurring in the pyramidal cell layer (Fig. 4*B*) (25). Next, extracellular field recordings were employed to assess basal synaptic transmission at the CA3 Schaeffer’s collaterals to CA1 synaptic inputs. Basal synaptic transmission at the Schaeffer’s collaterals to CA1 synaptic inputs remained unaffected by DM BDEVs signaling. Specifically, the input-output curve of the CA1 field excitatory post-synaptic potential (fEPSP) slope in response to the CA3 fiber stimulation under basal conditions showed no significant change between and receiving control BDEVs or DM BDEVs (control mean = 0.3 ± 0.1 mV/ms, n = 10 slices from animals receiving control BDEVs; DM mean = 0.3 ± 0.1 mV/ms, n = 12 slices from animals receiving DM BDEVs; *p* = 0.7; Fig. 4*C*). Similarly, DM BDEVs did not impact paired-pulse facilitation (control mean = 1.1 ± 0.1; DM mean = 1 ± 0.1 paired pulse ratio; *p* = 0.4; Fig. 4*D*), indicating the preservation of short-term Ca^2+^-dependent presynaptic function. Further investigation into synaptic plasticity was conducted by delivering tetanic stimulation (100 Hz for 1 s) at these synaptic inputs. Notably, while animals receiving control BDEVs displayed intact LTP, the group receiving DM BDEVs exhibited a complete loss of LTP (*p* < 0.0001; Fig. 4*E*). The fEPSP slope 60 min after tetanic stimulation was greatly potentiated in mice receiving control BDEVs (231.5 ± 20.1%) denoting the expected occurrence of LTP in response to stimulation. Strikingly, the fEPSP slope after tetanic stimulation remained unchanged after stimulation compared to the pre-tetanic baseline period in the mice receiving DM BDEVs (98.4 ± 4.9%, Fig. 4*E*) denoting lack of activity-dependent synaptic plasticity at CA3-CA1 inputs previously exposed to DM BDEVs. Notably, the lack of effect of DM BDEVs on basal synaptic transmission (Fig. 4*C*) and on paired-pulse facilitation (Fig. 4*D*) suggests that changes in long-term synaptic plasticity induced by DM BDEVs are likely driven by alterations in post-synaptic function. In summary, these findings demonstrate that exogenously delivered DM BDEVs have the capacity to impair synaptic function in naïve adult animals, which could potentially be explained by their differential proteome content.

### Exposure to LRRTM1 Disrupts Excitatory Synaptic Activity in CA1 Pyramidal Neurons

**Fig. 5:**
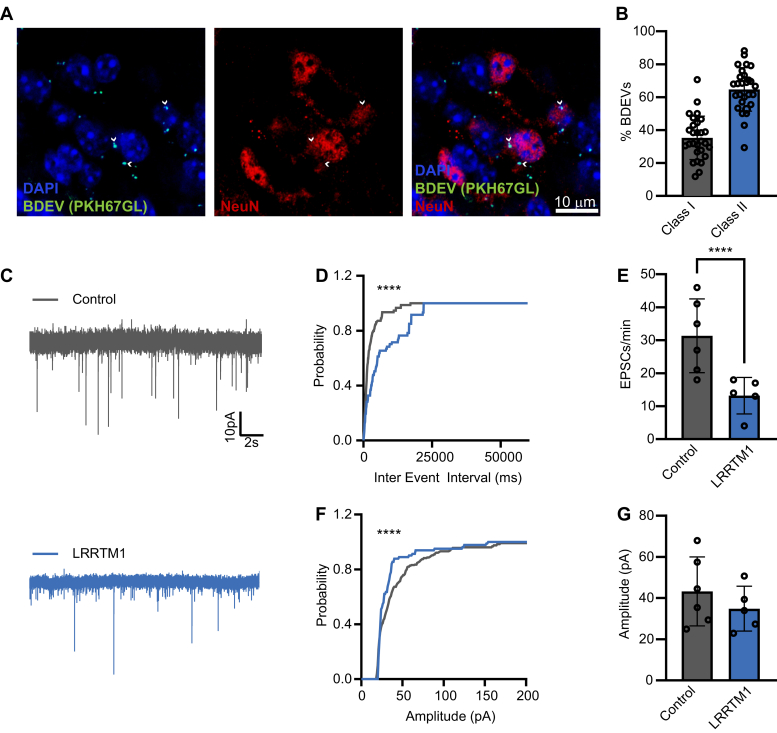
**Effects of LRRTM1 on EPSCs in CA1 pyramidal neurons.***A*, AiryScan image following injection of BDEVs (*Green*), DAPI (*blue*) and NeuN (*red*). *Arrows* indicate BDEVs imaged within close proximity to NeuN-labeled neurons. Scale bar indicates 10 μm. *B*, % of BDEVS near (Class I: *gray*) and at a distance from (Class II: *blue*) NeuN-labeled neurons compared to the total number of BDEVs. *C*, representative traces of sEPSCs recorded from CA1 pyramidal neurons from hippocampal slices treated with either vehicle (0.01% BSA, *gray*) or 100 ng/ml LRRTM1 (*blue*). LRRTM1 exposure results in a decrease in frequency of sEPSCs (*D* and *E*). This effect was accompanied by altered cumulative distribution of sEPSC amplitudes (*F*). Although this was effect was not observed in direct comparison of mean EPSC amplitudes (*G*). In (*E* and *G*), data points represent the total number of EPSCs observed during 1 min of recording (*E*) and the average amplitude of observed EPSCs (*G*) from each recording (control n = 6 and LRRTM1 n = 5). Data in (*C* and *E*) are mean ± SEM; ∗∗∗∗ indicates *p* < 0.01 as determined by an unpaired *t* test. Data in *D*, *F* are cumulative distributions of means; ∗∗∗∗ indicates *p* < 0.0001 as measured by a Kolmogorov-Smirnov test.

Having observed the effects of exogenously delivered DM BDEVs on long-term synaptic plasticity, we next sought to interrogate specific DEPs that may represent mechanistic drivers of these alterations. Critically, we first investigated the distribution of BDEVs in the hippocampus to verify that BDEVs deliver their contents to target cells. To do so, we imaged fluorescently labeled BDEVs in the hippocampus using AiryScan microscopy (Fig. 5*A*). Labeling of CA1 pyramidal neurons using NeuN showed that BDEVs fell into two broad categories: either within or near the NeuN-labeled neurons (Class I) or at a distance from the neurons (Class II; Fig. 5*B*), indicating that the effects of BDEVs from DM-exposed animals on target cells could be mediated through the delivery of DEPs within or near the NeuN-labeled neurons. One DEP of interest, which was found upregulated in DM BDEVs is leucine rich repeat transmembrane neuronal 1 (LRRTM1), a synaptogenic cell-adhesion protein that has been implicated in schizophrenia. LRRTM1 is a critical mediator of alpha-amino-3-hydroxy-5-methyl-4-isoxazole-propionate (AMPA) receptor-mediated excitatory transmission and has been implicated in LTP (45, 46). Thus, we hypothesized that the observed impairments in synaptic activity (Fig. 4*E*) resulting from DM BDEV exposure could be in part related to increased LRRTM1 in the hippocampus delivered by BDEVs. To test this hypothesis, acute hippocampal slices were prepared from naïve mice and treated with either vehicle (0.01% BSA) or 100 ng/ml LRRTM1. Then, whole-cell voltage clamp recordings were obtained to assess the frequency and amplitude of spontaneous excitatory postsynaptic currents (sEPSCs). Whole-cell patch-clamp recordings from CA1 pyramidal neurons (Fig. 5) demonstrated a significant reduction in the number of sEPSCs per minute following exposure to LRRTM1 (Fig. 5, *A*–*C*; 31.3 ± 4.6 EPSCs/min, n = 6 cells) compared to vehicle (13.2 ± 2.5; n = 5 cells, *p* = 0.0095). The reduction in sEPSC frequency was accompanied by a shift in the cumulative distribution of EPSC amplitude following treatment with LRRTM1 (Fig. 5*F*). However, a direct comparison of average sEPSC amplitudes following treatment with LRRTM1 (Fig. 5*G*; 43.3 ± 6.8 pA) compared to control (34.9 ± 4.8 pA, *p* = 0.3617) was not significant, indicating a less robust phenotype compared to sEPSC frequency. While further investigations are needed to elucidate the mechanism of LRRTM1 and other DEPs identified in this study, the observed electrophysiological effects support a direct role of DEPs from BDEVs in mediating DM-induced synaptic dysfunction.

## Discussion

Our study provides the following major findings: (i) early life DM exposure significantly alters proteome compositions in BDEVs; (ii) BDEVs from DM exposed mice selectively inhibit activity-dependent, long-term synaptic plasticity in the hippocampus of adult non-exposed subjects (*i.e.*, those with normal development) with no effects on basal synaptic transmission; (iii) The effects of DM BDEVs are measurable within only 36 h after injection suggesting readily inducible content in DM BDEVs; (*iv*) given that the differentially expressed proteome in DM BDEVs is largely associated with synaptic function, it is conceivable that the observed effect on synaptic plasticity is mediated by its proteome content.

Our proteomic analysis concluded that BDEV samples were consistent with previous studies in size, shape, concentration, and markers (20, 31). Additionally, the samples showed a low overall variability of protein changes within the group, meaning that the assay had the sensitivity to detect reasonable and physiologically relevant changes in protein levels. Results were internally consistent with the proteome of BDEVs and outwardly consistent with other studies of BDEV proteome (29).

The most striking finding of the GO overrepresentation analysis of DEPs is related to synapse and dendritic spine morphogenesis and development. Our results demonstrate the upregulation of various proteins, including neuroligin-3 (NLGN3), LRRTM1, and OPA1 mitochondrial dynamin like GTPase (OPA1), all of which play critical roles in dendritic spine morphogenesis and development. Additionally, OPA1 is involved in numerous pathways, potentially independent of NLGN3 and LRRTM1 (Fig. 3).

The discovery of NLGN3 aligns with previous findings indicating that neuroligins, a family of postsynaptic cell adhesion proteins, play a crucial role in synapse formation and brain function (47). Through interactions with their presynaptic binding partner, neurexin-3, neuroligins establish functional parameters of synapses by regulating the recruitment of synaptic proteins (48). While extensive studies have reported alterations in inhibitory synaptic transmission in knockout or knockdown models of NLGN3 there remains a gap in understanding how NLGN3 overexpression may lead to altered synaptic function (48, 49, 50). Furthermore, four splice isoforms of NLGN3 have been identified, each playing a different role in the excitatory and inhibitory synapse function (50). Since each isoform can form homodimers and heterodimers, overexpressing a particular NLGN3 isoform may plausibly induce a loss-of-function in dimer binding partners through a dominant negative effect contributing to the synaptic deficits induced by DM BDEVs. Future studies should investigate whether early life exposure to DM alters the expression of specific isoforms of NLGN3 and how these changes may impact synaptic function.

OPA1, which is a master regulator of mitochondrial dynamics, was also found to be upregulated in BDEVs from DM exposed animals (51). Notably, OPA1 also plays a critical role in neuronal maturation and plasticity (52) with haploinsufficiency altering dendritic spine density and morphology (52) and driving spatial memory deficits related to neurogenesis (52). It is possible that an imbalance in the equilibrium of OPA1 within mitochondrial dynamics, either alone or in combination with the overexpression of LRRTM1 and NLGN3, could lead to the loss of LTP observed in BDEVs from DM receiving mice.

Another striking finding was alterations in proteins associated with the phospholipase C (PLC) pathway also linked to OPA1. It has been reported that OPA1 regulates apoptosis by sequestering cytochrome c in mitochondrial cristae within this pathway, and its cleavage is regulated by the prohibitin (PHB) complex (53).

Two other key proteins in this pathway are PHB1 and PHB2. Interestingly, we observed an upregulation of PHBs in the BDEVs of DM-exposed animals. In neurons, PHB has a protective function against various stresses, with its deletion causing OPA1 destabilization and subsequent neurodegeneration. Conversely, increased PHB levels are believed to offer neuroprotection against OPA1 destabilization (53). Both PHB1 and PHB2 are mainly located in the mitochondrial inner membrane; however, they are also found in the plasma membrane where BDEVs are produced (54). PHB1 is upregulated in neurons experiencing stressors, including oxidative stress, electrical stimulation, hypoxia-ischemia, oxygen-glucose deprivation, exercise-induced neuroplasticity, and schizophrenia-related oligodendrocyte dysfunction (54). Here, we show a nearly 20-fold increase in PHB1, consistent with reports of oxidative stress caused by DM exposure (55). This suggests that early-life exposure to DM may induce a cellular stress response within the brain, which is reflected in the DM BDEV proteome, indicating another form of neurotoxicity.

LRRTM1, a postsynaptic cell adhesion protein, was found to be upregulated in the DM BDEVs proteome (Fig. 2*B*). This protein is essential for LTP at CA3-CA1 synapses and contributes to hippocampal and dentate gyrus circuit (56). Knockout studies of LRRTM1/2 in neurons show reduced spine density, suppression of AMPA receptor-mediated EPSCs and impaired LTP in the hippocampus (46). Given LRRTM1’s key role in hippocampal synaptic transmission and plasticity (46, 56), we hypothesized that LTP impairments might result from disrupted LRRTM1 function. In our study, exogenously delivered LRRTM1 produced effects consistent with prior research on LRRTM1 genetic manipulation, suggesting that excess LRRTM1 interferes with the native protein's role in organizing excitatory synapses. Like neuroligins, LRRTM1/2 forms homo- and heterodimers, raising the possibility that its upregulation may cause a dominant negative effect, similar to neuroligins, contributing to the loss of LTP observed in mice treated with BDEVs from DM-exposed mice (57). Although the exact mechanism by which exogenous LRRTM1 impairs excitatory synaptic function requires further investigation, our results highlight the potential for DEPs in DM BDEVs to disrupt excitatory transmission.

Another relevant protein involved in regulating the PLC pathway is adenylate cyclase activating polypeptide receptor type 1 (ADCYAP1R1, also known as PAC1R), which serves as a receptor for adenylate cyclase activating polypeptide 1 (58). ADCYAP1R1 was found upregulated in DM BDEVs. Adcyap1r1 RNA and its ligand, pituitary adenylate cyclase activating peptide, were found to be upregulated in the prefrontal cortex of schizophrenia (SCZ) patients (58) which is a potential association between the PLC pathway, neurodevelopmental disorders, and early life DM exposure.

We also saw robust changes in mitochondrial proteins directly related to ATP synthesis, such as ATP5D, which is upregulated in individuals with Parkinson’s disease compared to middle-aged control individuals (59). While our focus was on the effects of DM from early-life exposure, it remains unclear whether DM exposure could lead to an increased risk of neurodegenerative disorders later in life. This aspect should be thoroughly investigated in future studies.

Additionally, while our results clearly demonstrate that exposure to DM alters the proteome of BDEVs, it is not yet known whether this is a result of changes to the overall brain proteome or changes in BDEV packaging, which should also be the focus of future experiments.

Overall, we conclude that the altered content of BDEVs from DM exposure is sufficient to cause dysregulation of multiple signaling pathways within the brain. Proteomic results provide several promising avenues for future research into the molecular mechanism whereby BDEVs from DM exposure inhibit hippocampal synaptic plasticity. This BDEV-mediated neurotoxicity is alarming, as it occurs within the NOAEL set by the EPA, which raises significant concerns about the risk of exposure in the general population and the potential correlation between pyrethroid exposure and the alarming increased incidence of ADHD, ASD, and other neurodevelopmental and neurodegenerative disorders.

While our studies focus on the brain, and specifically the hippocampus, it is crucial to note that BDEVs travel throughout the brain and thus will affect multiple brain regions beyond the hippocampus. Additionally, evidence exists for extracellular vesicles, not of brain origin, to travel throughout the body raising the possibility of multiorgan effects and crosstalk between the brain and peripheral organ axis. By elucidating the distinct proteome content in DM BDEVs, these studies could serve to define biomarkers of exposure and contribute to defining each individual’s exposome for assessing disease risk (60).

## Data Availability

All data will be made available upon request. Please contact Dr Fernanda Laezza (felaezza@utmb.edu) for data.

## Supplemental data

This article contains supplemental data.

## Conflict of interests

The authors declare that they have no conflicts of interest with the contents of this article.
