# Supplementary material for: Early Life Exposure to Deltamethrin Impairs Synaptic Function by Altering the Brain-Derived Extracellular Vesicle Proteome
Source: Mol Cell Proteomics. 2024 Dec 31;24(2):100902. doi: 10.1016/j.mcpro.2024.100902 (PMC11847076; doi:10.1016/j.mcpro.2024.100902)
Supplement: Supplemental material [file mmc1.pdf]

Supplemental Table 1. Primary and secondary Antibody (Ab) used for western blot analysis.

Antibody (Cat#)	MW (kDa)	Running condition	Host	1^0 Ab dilution	2^0 Ab	2^0 Ab dilution
GM130 (610822)	130		Mouse	1:1000	HRP-conjugated rabbit anti-mouse IgG	1:10,000
Alix (H-270) (sc-99010)	95	Reducing	Rabbit	1:500	HRP-conjugated goat anti-Rabbit	1:10,000
Hsp-70 (SAB4200714)	70	Reducing	Mouse	1:1000	HRP-conjugated rabbit anti-mouse IgG	1:10,000
Beta-Actin (AM1829B)	41	Reducing	Mouse	1:1000	HRP-conjugated rabbit anti-mouse IgG	1:10,000
CD81 (MCA1846)	26	Non-reducing	Hamster	1:500	HRP-conjugated goat anti-Hamster	1:10,000

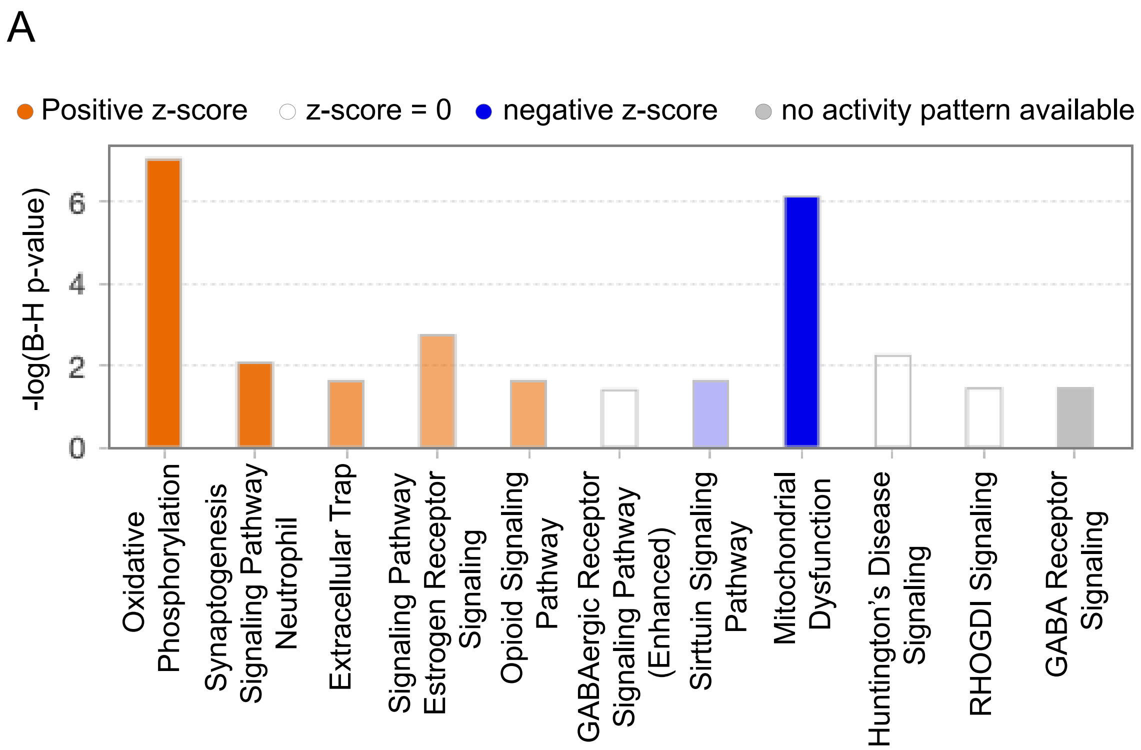

Supplemental Figure 1. IPA pathway analysis

A. IPA analysis of the positive/activated (orange) and negative/inhibited (blue) pathways found to be altered in the DM proteome.
